# RNAi silencing of the *SoxE* gene suppresses cell proliferation in silkworm BmN4 cells

**DOI:** 10.1007/s11033-014-3348-6

**Published:** 2014-04-11

**Authors:** Ling Wei, Zhiqing Li, Daojun Cheng, Takahiro Kusakabe, Minhui Pan, Jun Duan, Yonghu Wang, Cheng Lu

**Affiliations:** 1 State Key Laboratory of Silkworm Genome Biology, Southwest University, Chongqing, China; 2 School of Life Science, Southwest University, Chongqing, China; 3 Laboratory of Silkworm Science, Kyushu University Graduate School of Bioresource and Bioenvironmental Sciences, Fukuoka, Japan

**Keywords:** Silkworm, SoxE, RNAi, Cell proliferation, Target

## Abstract

The transcription factor SoxE is mainly expressed in the gonad and involved in the regulation of gonad development and sex determination in animals. Here, we used the silkworm ovary-derived BmN4-SID1 cell line to survey the roles of the silkworm SoxE protein (BmSoxE) and predict its candidate binding targets. RNAi-mediated silencing of *BmSoxE* expression suppressed cell proliferation in BmN4-SID1 cells. A further cell cycle analysis revealed that this inhibition of cell proliferation was largely due to cell cycle arrest in G1 phase when *BmSoxE* expression was blocked in BmN4-SID1 cells. Genome-wide microarray expression analyses demonstrated that the expression levels of a set of genes were significantly altered following *BmSoxE* RNAi. More than half of these genes contained conserved binding sites for HMG box domain of the Sox proteins and were predicted to be candidate binding targets for BmSoxE. Importantly, some of the candidate targets may be associated with the effect of BmSoxE on cell proliferation. Several candidate target genes showed gonad-specific expression in silkworm larvae. Taken together, these data demonstrate that BmSoxE is required for cell proliferation in silkworm BmN4-SID1 cells and provide valuable information for further investigations of the molecular control exerted by the BmSoxE protein over cell proliferation and gonad development in the silkworm.

**Electronic supplementary material:**

The online version of this article (doi:10.1007/s11033-014-3348-6) contains supplementary material, which is available to authorized users.

## Introduction

The Sox transcription factor family has been well studied in animals and demonstrated to be involved in various physiological processes, including sex determination, gonad development, embryogenesis, nervous system development, and chondrogenesis [1, 2]. Structurally, each Sox protein contains a highly conserved high-mobility group box (HMG box) domain, which is required for the recognition and binding of a conserved DNA motif, (A/T)(A/T)CAA(A/T)G, in the upstream untranslated region (UTR) of its target genes. A series of studies have demonstrated that Sox proteins function as either activators or repressors to activate or inhibit the transcription of their targets, respectively [3, 4]. Evolutionarily, the Sox proteins found in animals can be subdivided into 10 groups, designated A to J, based on their sequence similarities [5].

The group E Sox proteins (hereafter, SoxE proteins) have been comprehensively studied. In mammals, the SoxE proteins include three members: Sox8, Sox9, and Sox10, which are involved in multiple developmental programs, such as testis development, sex determination, and nervous system development [2]. Recently, several reports have focused on the identification of binding targets of the SoxE proteins. For example, in mice, Sox9 was observed to activate the transcription of *Amh* and *Vanin*-*1* during testis development [6], and *Col2a1* during chondrogenesis [7]. Sox10 in mice can regulate the expression of *Connexin32* and *Connexin47* in oligodendrocytes during myelination [8] and that of *MEF2C* during melanocyte development [9]. The direct transcriptional targets of Sox10 include genes encoding proteolipid protein, extracellular superoxide dismutase, and pleiotrophin in rat Schwannoma cells [10]. Moreover, genome-wide analysis has revealed hundreds of genes that are potential binding targets for Sox9 and/or Sox8 in mice and rats [11, 12]. Because of the functional redundancy of the different SoxE proteins in mammals [13], it may be difficult to determine their targets.

Among insects, homologs of the mammalian SoxE proteins have been identified in *Drosophila melanogaster*, *Apis mellifera*, *Tribolium castaneum*, *Anopheles gambiae*, and *Bombyx mori* [14–18]. One member of the SoxE protein family has been found in insects, with the exception of *A*. *mellifera*, which exhibits two group E genes that most likely arose through gene duplication in an ancestral lineage [17, 18]. A previous study in *D. melanogaster* confirmed that SoxE mutations affect the proper morphogenesis of the testis during the pupal stage and markedly reduce the size of the adult testis [19]. More importantly, the replacement of mouse Sox10 with *D. melanogaster* SoxE was able to rescue neural crest and oligodendrocyte development [20], revealing conserved roles of the SoxE proteins between vertebrates and invertebrates. However, the signaling pathways and functions of insect SoxE proteins remain poorly understood. In particular, no identified binding targets of insect SoxE proteins have been reported, either at the individual or cellular level.

The silkworm (*B. mori*) is an excellent model for studying insect biology [21]. We previously observed that the silkworm *SoxE* (*BmSoxE*) gene is highly expressed in the gonad [14]. Recently, the whole-genome sequence and genome-wide microarray expression data for the silkworm are available [22, 23]. Additionally, the silkworm ovary-derived BmN4-SID1 cell line, which harbors the *SID1* gene from *Caenorhabditis elegans* that shows an increased efficiency in the uptake of extracellular double-stranded RNA (dsRNA) in the RNA interference (RNAi) analysis of genes of interest, has been established [24]. In this study, we performed RNAi-mediated knockdown of *BmSoxE* expression in BmN4-SID1 cells and observed that BmN4-SID1 cells were markedly compromised in terms of cell proliferation and cell cycle progression following this procedure. Microarray analysis demonstrated that the expression of numerous genes was down- or up-regulated following *BmSoxE* RNAi. A portion of these genes containing binding motifs for the HMG box domain of the Sox protein were considered as candidate targets of the BmSoxE protein and may be involved in the BmSoxE-mediated regulation of cell proliferation.

## Materials and methods

### Cell lines

The cultured silkworm ovary-derived BmN4 cell line and the BmN4-SID1 transgenic cell line were used in our experiment [24]. The BmN4 cell line was derived from the silkworm ovary and used to examine subcellular localization of the BmSoxE protein and profile *BmSoxE* expression. The BmN4-SID1 cell line was established via introduction of the *C*. *elegans* *SID1* gene, which can greatly enhance the uptake of dsRNA from host cells into BmN4 cells [24]. Thus, the BmN4-SID1 cell line has been shown to possess high efficiency in the uptake of exogenous dsRNA [24]. The BmN4-SID1 cell line was used to perform RNAi knockdown of the *BmSoxE* gene. The BmN4 and BmN4-SID1 cell lines were maintained at 27 °C in IPL-41 medium (Sigma, USA) supplemented with 10 % fetal bovine serum (Life Technologies, USA).

### *BmSoxE* expression profiling and subcellular localization in BmN4 cells

A semi-quantitative RT-PCR (reverse transcription-polymerase chain reaction) experiment was performed to determine whether the *BmSoxE* gene was expressed in BmN4 cells. Extraction of total RNA, cDNA synthesis, and RT-PCR analysis were performed according to a previously described procedure [25]. The silkworm *glyceraldehyde*-*3*-*phosphate dehydrogenase* (*BmGAPDH*) gene was selected as a control. The primers employed in the analysis are provided in Online Resource 1.

For the analysis of subcellular localization, the open reading frame (ORF) of the *BmSoxE* gene was cloned into the pENTR™11 vector (Invitrogen) to construct an entry plasmid. The nucleotide sequence of the plasmid was confirmed via DNA sequencing. Then, the entry plasmid harboring the *BmSoxE* gene was used to construct a destination vector with the pi2VW plasmid containing Venus fluorescence protein via a Gateway reaction [26]. BmN4 cells were transfected with 100 ng of the expression plasmid harboring the Venus-fused *BmSoxE* gene. On the 3rd day after transfection, the treated cells were seeded onto a cover slip coated with poly-l-lysine, fixed with 3.7 % formaldehyde in phosphate-buffered saline (PBS) for 10 min, and permeabilized with 0.1 % Triton X-100 in PBS for 5 min. Cellular DNA was stained with DAPI (Invitrogen, USA). Finally, light and fluorescence microscopy images were captured using an Olympus DX51 microscope (Olympus, Japan).

### RNAi knockdown of *BmSoxE* expression in BmN4-SID1 cells

The synthesis of dsRNAs targeting *EGFP* (enhanced green fluorescent protein; dsEGFP) or *BmSoxE* (dsBmSoxE) and the dsRNA treatment of BmN4-SID1 cells were performed according to a previously described protocol [26]. We collected BmN4-SID1 cells at different time points, including the 1st, 3rd, 5th, and 7th days after dsBmSoxE or dsEGFP treatment, for further analysis.

### Cell proliferation assay

For cell proliferation assays, approximately 3.0 × 10^3^ BmN4-SID1 cells were seeded into 96-well plates and cultured in a final volume of 100 μl of IPL-41 medium. dsBmSoxE or dsEGFP (control) were added to the medium at a final concentration of 0.5 μg/ml. The cells were labeled with 10 μl of WST-8 solution (Cell counting Kit-8; Dojindo) for 12 h before the indicated time points, including the 1st, 3rd, 5th, and 7th days after dsRNA treatment.

The absorbance was measured at 450 nm in a 96-well spectrophotometric plate reader according to the manufacturer’s protocol, and proliferation curves were plotted using the absorbance at each time point. All of the experiments were performed in triplicate. The data were compared between the treated and the corresponding control groups using Student’s *t* test, and a p value <0.05 was considered statistically significant.

### Flow cytometry assay

To analyze the effect of *BmSoxE* RNAi on the cell cycle, the cell cycle distribution was determined by measuring the cellular DNA content using a flow cytometer according to a previously described procedure [24].

### Microarray analysis

BmN4-SID1 cells were cultured in IPL-41 medium to which dsBmSoxE or dsEGFP were added and harvested after 7 days of incubation. Total RNA was then isolated using TRIzol reagent (Invitrogen, USA). Approximately 1 μg of RNA from each sample was subjected to reverse transcription using M-MLV Reverse Transcriptase according to the manufacturer’s instructions (Promega, USA). The efficiency of the knockdown of the *BmSoxE* gene was evaluated via RT-PCR using specific primers as described in Online Resource 1. The *BmGAPDH* gene was employed as an endogenous control.

For the microarray experiment of gene expression profiling, the hybridization and data acquisition were performed by CapitalBio Corp (China). Three biological replicates were conducted. Raw microarray data were normalized according to a previously described method [23]. A gene was considered to be expressed in any treatment if its signal intensity exceeded signal intensity units of 200 after subtracting the background and normalizing the raw microarray data. The fold change in expression level for a gene following *BmSoxE* RNAi was calculated by comparing the normalized expression intensity of a gene after *BmSoxE* RNAi to the intensity of the same gene following *EGFP* RNAi. The significance (p value) of the expression change in a gene was evaluated using paired *t*-test and further adjusted using the Benjamini-Hochberg method [27–29]. Finally, a gene was defined as a candidate for being significantly down- or up-regulated if its change in expression level was greater than 2.0-fold (i.e., showing an intensity ratio less than 0.5 or greater than 2.0) with a p value <0.05. All of the microarray data presented in this study have been deposited in the GEO database under accession number GSE53240.

For validation of the microarray data, we randomly selected five down-regulated and five up-regulated genes following *BmSoxE* RNAi from the list provided in Online Resource 2 and performed RT-PCR experiments. The cDNA templates subjected to RT-PCR were identical to those employed in the microarray analysis. The primers are listed in Online Resource 1.

The tissue-specific expression patterns of the genes that were down- or up-regulated after *BmSoxE* RNAi were profiled based on the microarray expression data in multiple tissues in silkworm larvae on 3rd day of the 5th instar [23].

The online program WEGO (http://wego.genomics.org.cn/cgi-bin/wego/index.pl) [30] was used to perform GO (Gene Ontology) annotations of functional categories for the selected genes.

### Searching for conserved binding sites of the HMG box domain

We fetched the sequences from the approximately 2.5 kb upstream UTR regions of the translation initiation sites of genes showing altered mRNA expressions following *BmSoxE* RNAi in BmN4-SID1 cells. These sequences were subjected to search for the conserved recognition and binding motif of the HMG box of Sox proteins using the online MatInspector program (http://www.genomatix.de; Core similarity threshold 0.8), which is a tool that searches for the binding sites of transcription factors [31]. The online WebLogo program (http://weblogo.berkeley.edu/) was used to display consensus binding sequences [32].

## Results

### BmSoxE localized to the nuclei of BmN4 cells

To determine the subcellular localization of the BmSoxE protein in silkworm ovary-derived BmN4 cells, we carried out transient expression of the BmSoxE protein fused to the C-terminus of Venus fluorescence protein. The result showed that the BmSoxE protein localized to cell nuclei (Online Resource 3a), which is consistent with its transcription factor activity. To examine the roles of the *BmSoxE* gene in silkworm ovary-derived BmN4 cells, we first checked whether the *BmSoxE* gene was expressed in BmN4 cells. RT-PCR results demonstrated that *BmSoxE* expression could be detected in BmN4 cells (Online Resource 3b).

### *BmSoxE* RNAi suppressed cell proliferation in BmN4-SID1 cells

**Fig. 1:**
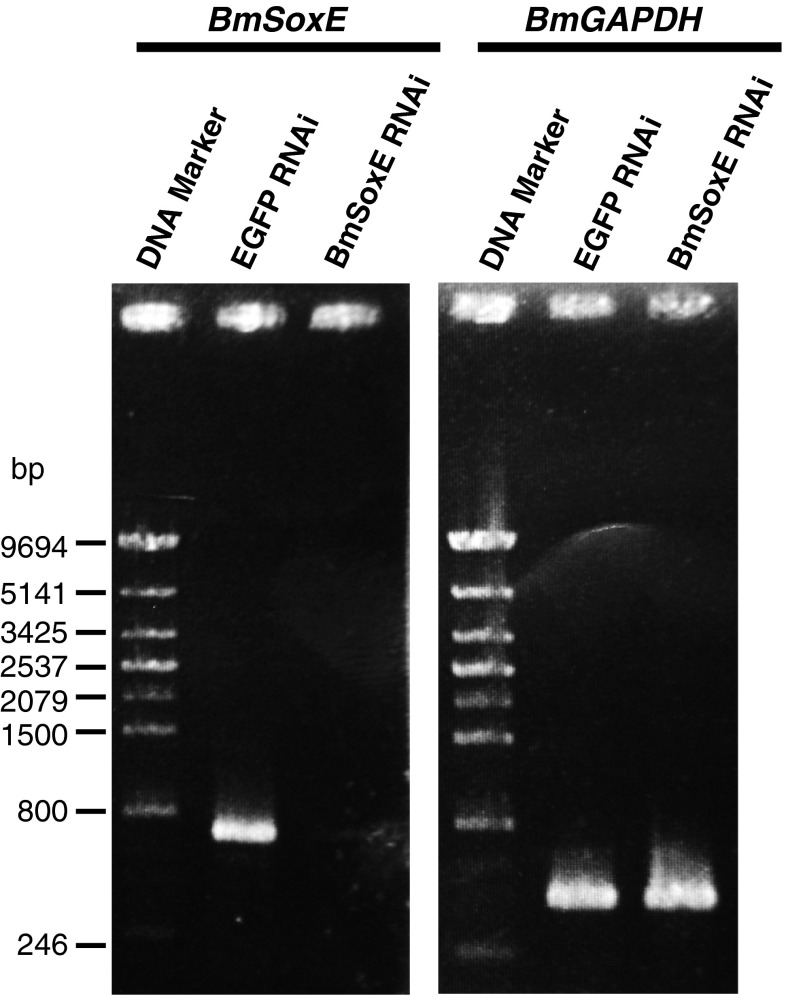
RNAi-based knockdown efficiency of *BmSoxE* expression in silkworm BmN4-SID1 cells. A significant reduction of *BmSoxE* expression occurred in BmN4-SID1 cells following *BmSoxE* RNAi compared with *EGFP* RNAi (control). *BmGAPDH* expression was used as a control

We performed a series of dsRNA-mediated RNAi experiments examining the *BmSoxE* gene in BmN4-SID1 cells to assess the effects of the knockdown of *BmSoxE* expression on cell growth. dsBmSoxE and dsEGFP (control) were introduced separately into BmN4-SID1 cells, and subsequent RT-PCR analysis demonstrated that *BmSoxE* expression was completely silenced in the BmN4-SID1 cells on the 7th day after dsBmSoxE treatment (Fig. 1).

**Fig. 2:**
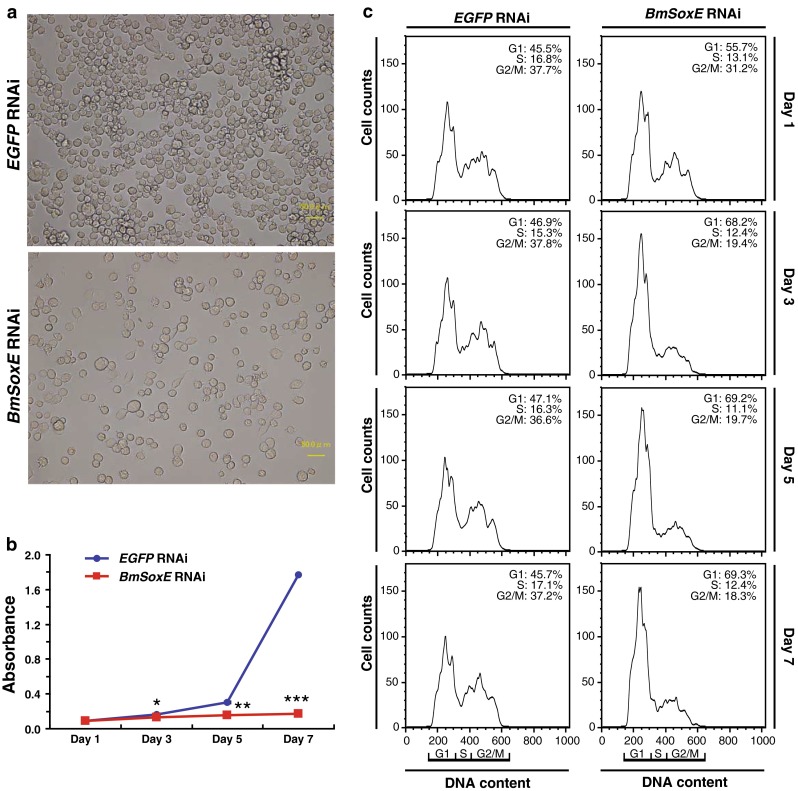
Effects of *BmSoxE* RNAi on cell proliferation and cell cycle progression in silkworm BmN4-SID1 cells. **a** On the 7th day after *BmSoxE* RNAi in silkworm BmN4-SID1 cells, the number of cells was markedly reduced compared with the *EGFP* RNAi as control. *Scale bar*: 50 μm. **b** Cell proliferation curves for BmN4-SID1 cells following *BmSoxE* RNAi at the indicated time points. Data are displayed as the mean ± SD of three independent experiments, *P < 0.05; **P < 0.01; ***P < 0.001, compared with the corresponding control. **c** Flow cytometry analysis of the time-course distribution of the cell cycle following *BmSoxE* RNAi

Intriguingly, the number of BmN4-SID1 cells decreased markedly on the 7th day after *BmSoxE* RNAi compared with BmN4-SID1 cells treated with dsEGFP (Fig. 2a). To analyze the effects of *BmSoxE* RNAi on cell proliferation in further detail, we collected BmN4-SID1 cells at different time points, including the 1st, 3rd, 5th, and 7th days after *BmSoxE* RNAi. The cell proliferation curves depicted in Fig. 2b revealed that *BmSoxE* RNAi began to suppress cell proliferation on the 3rd day, and significant inhibition was achieved on the 7th day, consistent with the findings presented in Fig. 2a.

To investigate whether the silencing of *BmSoxE* expression affected cell cycle progression, we harvested BmN4-SID1 cells at the indicated time points after incubating them with dsBmSoxE or dsEGFP and performed flow cytometry analysis. Compared with *EGFP* RNAi, the number of BmN4-SID1 cells in G2/M phase was decreased by approximately 18 % on the 3rd day after *BmSoxE* RNAi, followed by an increase of approximately 22 % in the number of cells at G1 phase and a decrease of approximately 4 % at S phase (Fig. 2c). This cell cycle arrest at the G1/S phases via *BmSoxE* RNAi continued until the 7th day, consistent with the observations from the cell proliferation curves.

### Genome-wide gene expression was altered after *BmSoxE* RNAi in BmN4-SID1 cells

**Fig. 3:**
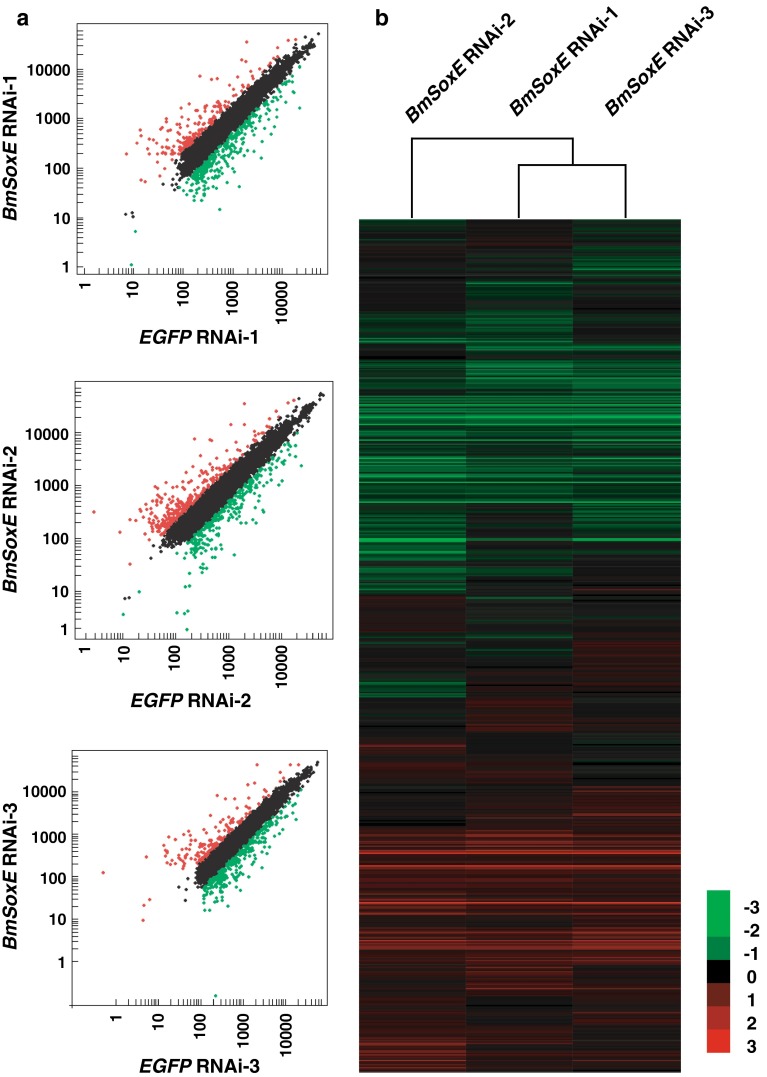
Genome-wide changes in gene expression following *BmSoxE* RNAi in silkworm BmN4-SID1 cells. **a** Expression intensity-based scatter plots of gene expression changes after *BmSoxE* RNAi in silkworm BmN4-SID1 cells. **b** Ratio-based hierarchical clustering of gene expression changes after *BmSoxE* RNAi

We used silkworm genome-wide expression microarray to profile gene expression changes in BmN4-SID1 cells on the 7th day after *BmSoxE* RNAi. The results showed that 6,195 and 6,188 genes were expressed in BmN4-SID1 cells associated with *BmSoxE* RNAi and *EGFP* RNAi, respectively. Summarily, a total of 6,275 genes were expressed in both RNAi experiments. The scatter plots (Fig. 3a) and clustering patterns (Fig. 3b) of gene expression obtained from three biological replicates displayed high similarities.

**Fig. 4:**
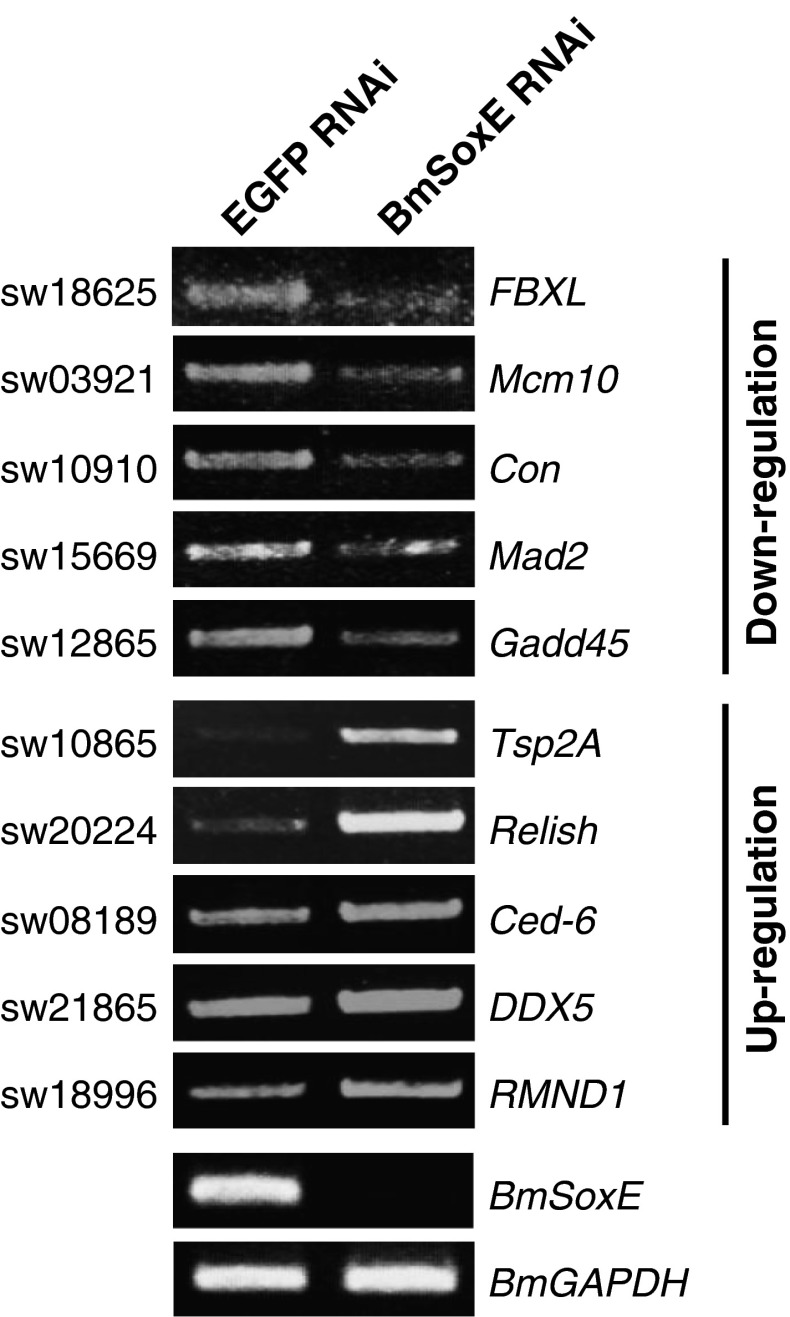
RT-PCR-based expression profiling of several differentially expressed genes, RT-PCR experiments were performed to validate expression changes of ten genes that were differentially expressed following *BmSoxE* RNAi, including five down-regulated genes and five up-regulated genes. *BmGAPDH* expression was used as a control

Compared with *EGFP* RNAi as the control, the expression levels of 320 genes were significantly altered following *BmSoxE* RNAi (Online Resource 2). Among these differentially expressed genes, 118 were down-regulated, and 202 were up-regulated. As expected, the *BmSoxE* gene was also included in the down-regulated gene list, further revealing a high efficiency of *BmSoxE* RNAi in BmN4-SID1 cells. Moreover, we arbitrarily selected ten differentially expressed genes to perform RT-PCR confirmation. Consistent with the microarray data, the expression levels of most of these tested genes were validated as being down- or up-regulated following *BmSoxE* RNAi (Fig. 4), compared with *EGFP* RNAi as the control.

Homologous annotation revealed that 88 down- and 140 up-regulated genes presented hits that were homologous to known genes or domains (Online Resource 2). Of the down-regulated genes, 16 displayed at least tenfold changes in expression level, which included genes encoding protein-glutamine gamma-glutamyltransferase, beta-ureidopropionase, gag protein, connectin, nervous system antigen 2, SEC14, retinol dehydrogenase 12, and SoxE. In addition, one of the six up-regulated genes that showed a greater than tenfold change in expression level was annotated as tetraspanin 2A.

**Fig. 5:**
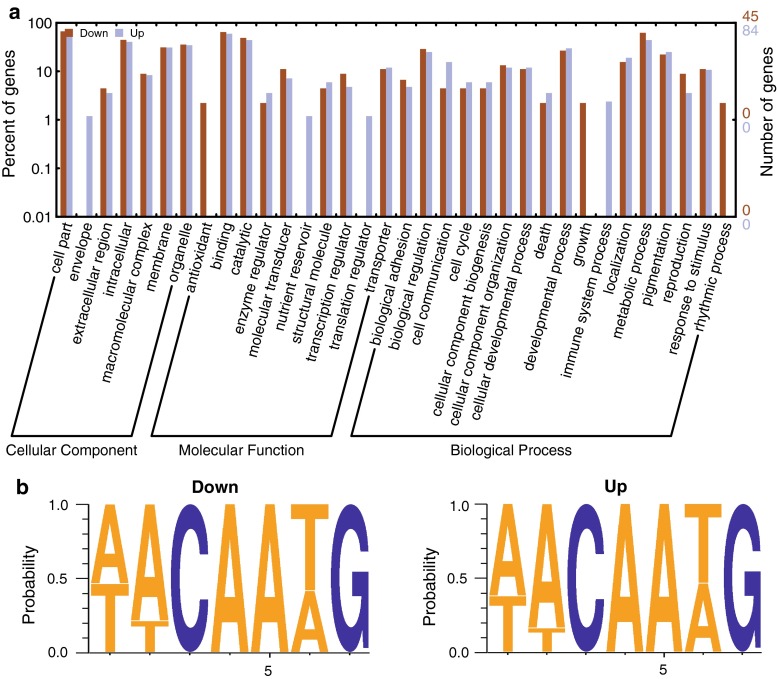
GO categories of differentially expressed genes and the conserved Sox protein binding motifs in their upstream UTR regions. **a** GO annotation of genes that were differentially expressed after *BmSoxE* RNAi was performed using the online WEGO program. **b** Multiple sequence display of conserved Sox protein binding motifs within the upstream regions of differentially expressed genes was generated with WebLogo

GO annotation of functional categories revealed that the genes expressed in BmN4-SID1 cells following *BmSoxE* RNAi or *EGFP* RNAi mainly possess catalytic and binding activities and are involved in development, metabolism, coloring, and other biological processes (Online Resource 4). Further comparative analysis indicated that several GO categories were specifically down-regulated following *BmSoxE* RNAi, such as antioxidants (peroxidasin, BGIBMGA000553) among molecular functions, and rhythmic processes (HLF protein, BGIBMGA003874) as well as growth (expanded protein, BGIBMGA010558) among biological processes, as shown in Fig. 5a. In contrast, several GO categories were particularly up-regulated, such as the nutrient reservoir (arylphorin alpha subunit, BGIBMGA009027) as well as translation regulator (EFTUD1, BGIBMGA001523) among molecular function and the immune system process category (collier, BGIBMGA000883; cuticular protein, BGIBMGA001862) among biological processes.

### Differentially expressed genes containing the conserved binding motif for the HMG box were considered as candidate BmSoxE binding targets

We fetched the sequences of the 2.5 kb upstream UTR regions of the translation initiation sites of the differentially expressed genes following *BmSoxE* RNAi and searched for the conserved recognition and binding sites for the HMG box of Sox proteins using the MatInspector program. As a result, we identified binding sites for the HMG box within the upstream UTRs of 108 differentially expressed genes, including 42 down-regulated genes and 66 up-regulated genes, of which 46 genes possessed at least two binding sites (Online Resource 2). These differentially expressed genes containing conserved binding motif for the HMG box were considered as candidate targets of the BmSoxE protein.

We extracted the consensus sequences of the binding sites for the HMG box from the upstream UTRs of candidate BmSoxE targets to check their base components using the online WebLogo program. As shown in Fig. 5b, the core bases of CAA and G were the same within all of HMG box binding motifs in the upstream UTRs of both down- and up-regulated genes.

### A set of candidate BmSoxE targets were related to cell proliferation

Given that the RNAi-based silencing of *BmSoxE* expression suppressed cell proliferation in BmN4-SID1 cells, we searched candidate BmSoxE targets that likely contribute to the regulation of cell proliferation based on homologous annotation. Notably, the homologs of 10 significantly down-regulated genes following *BmSoxE* RNAi found in other organisms have been confirmed to be involved in the regulation of cell proliferation, which included *MU2*, *Glypican*, *Mad2*, *DENN*, *Gadd45*, *GlcT*-*1*, *Mcm10*, *TACC*, *Peroxidasin*, and *Nudt1* (Table 1). In addition, we observed that the homologs of nine significantly up-regulated genes are also associated with cell proliferation, which included *Tsp2A*, *SEPW1*, *Ced*-*6*, *TBC1D7*, *Cyt*-*b5*, *Tensin*, *Orb2*, *Vein*, and *Cyt*-*c*-*p* (Table 1).

**Table 1:** Candidate BmSoxE targets related to cell proliferation

Categories	Probe	Gene ID	Gene symbol	Gene description	Ratio	P value	Number of binding sites for HMG box
Down-regulation	sw20407	BGIBMGA008152	*MU2*	Mutator 2	0.15	0.00155	1
	sw03953	BGIBMGA003353	*Glypican*	Glypican	0.34	0.00212	2
	sw15669	BGIBMGA012734	*Mad2*	Mitotic spindle assembly checkpoint protein mad2	0.37	0.04374	1
	sw17999	BGIBMGA011241	*DENN*	DENN domain-containing protein	0.37	0.04842	1
	sw12865	BGIBMGA013938	*Gadd45*	Growth arrest and DNA damage-inducible protein GADD45 alpha	0.37	0.00207	1
	sw00999	BGIBMGA009686	*GlcT*-*1*	Glucosylceramide synthase	0.41	0.01086	1
	sw03921	BGIBMGA007331	*Mcm10*	Sensitized chromosome inheritance modifier 19	0.44	0.01286	2
	sw04367	BGIBMGA005038	*TACC*	Transforming acidic coiled-coil-containing protein	0.44	0.00773	1
	sw08623	BGIBMGA000553	*Peroxidasin*	Peroxidasin	0.44	0.02598	5
	sw13301	BGIBMGA005699	*Nudt1*	7,8-dihydro-8-oxoguanine triphosphatase	0.48	0.04450	1
Up-regulation	sw10865	BGIBMGA001022	*Tsp2A*	Tetraspanin 2A	19.57	0.00877	1
	sw14661	BGIBMGA010104	*SEPW1*	Thioredoxin-like protein	4.62	0.00003	1
	sw08189	BGIBMGA009770	*Ced*-*6*	Ced-6	3.52	0.00271	5
	sw07012	BGIBMGA010784	*TBC1D7*	TBC1 domain family, member 7	3.36	0.02036	3
	sw09668	BGIBMGA003014	*Cyt*-*b5*	Cytochrome b5	3.05	0.02440	1
	sw02970	BGIBMGA013563	*Tensin*	Tensin	2.61	0.03828	3
	sw19380	BGIBMGA000174	*Orb2*	Orb2	2.26	0.00240	1
	sw12509	BGIBMGA012742	*Vein*	Vein	2.15	0.00441	1
	sw15825	BGIBMGA009012	*Cyt*-*c*-*p*	Cytochrome *c* proximal	2.05	0.00023	2

### Core genes involved in cell cycle regulation displayed no significant expression changes following *BmSoxE* RNAi

Curiously, we observed that most of the well-studied core cell cycle regulators involved in cell cycle progression and DNA replication, which are two cell cycle processes orchestrating cell proliferation, were excluded from the collection of differentially expressed genes after *BmSoxE* RNAi (Table 1). The two exceptions to this pattern were *Gadd45* and *Mcm10*, which are core regulators that were significantly down-regulated after *BmSoxE* RNAi (Table 1). Further analysis indicated that most of the core cell cycle regulators exhibited no significant expression changes of less than 2.0-fold, and some regulators also possessed HMG box binding sites within their upstream UTR regions (Online Resource 5 and Online Resource 6). Among the cell cycle progression-related genes showing detectable expression levels in BmN4-SID1 cells associated with *BmSoxE* RNAi or *EGFP* RNAi, seven (i.e., *Myc*, *skp2*, *p27*, *cyclin B*, *E2F transcription factor 4*-*like protein*, *cdc2*-*related kinase*, and *ras*) and three genes (i.e., *cyclin*-*dependent kinase regulatory* *subunit*, *cdc25*-*like protein*, and *cyclin A*) exhibited relatively greater down-regulation and up-regulation, respectively, following *BmSoxE* RNAi (Online Resource 5).

Additionally, we surveyed the expression patterns of core genes related to DNA replication and observed that among the expressed DNA replication-related genes, eight genes (i.e., *Orc2*, *RfC3*, *Mcm5*, *RfC4*, *RPA70*, *Mcm6*, *Mcm8*, and *Mcm3*) displayed moderate down-regulation after *BmSoxE* RNAi, whereas *cyclin A* exhibited moderate up-regulation (Online Resource 6).

### Candidate BmSoxE targets were expressed in the silkworm gonad

**Fig. 6:**
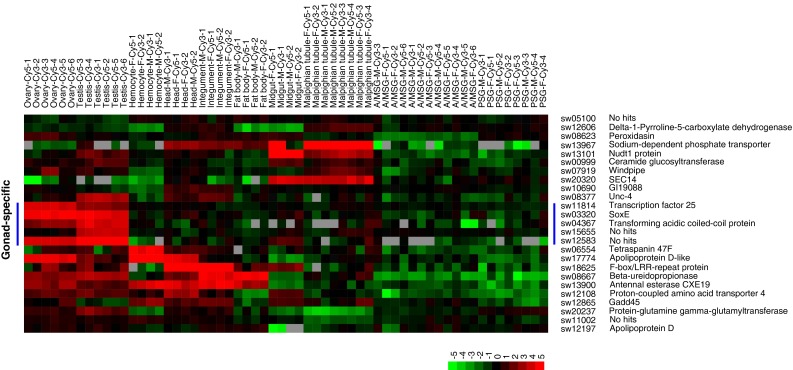
Larval tissue-specific expression patterns of candidate BmSoxE targets that were down-regulated following *BmSoxE* RNAi in silkworm BmN4-SID1 cells. The original microarray data used for expression profiling of candidate BmSoxE targets were derived from previous reports examining gene expression in multiple tissues of silkworm larvae (GSE17571). A/MSG, anterior/median silk gland. *PSG* posterior silk gland, *F* female, *M* Male, *Cy5* red-fluorescent dye, *Cy3* green-fluorescent dye. *Arabic numerals* represent the number of the biological replicates

**Fig. 7:**
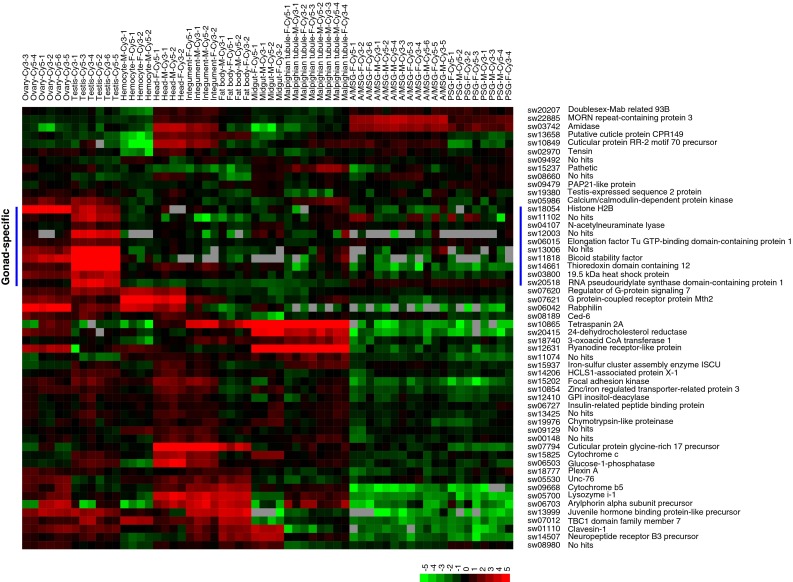
Larval tissue-specific expression patterns of candidate BmSoxE targets that were up-regulated following *BmSoxE* RNAi in silkworm BmN4-SID1 cells. A/MSG, anterior/median silk gland. *PSG* posterior silk gland, *F* female, *M* Male, *Cy5* red-fluorescent dye, *Cy3* green-fluorescent dye. *Arabic numerals* represent the number of the biological replicates

*BmSoxE* expression has been confirmed to be enriched in the silkworm gonad [14]. Based on the analysis of microarray data of genome-wide gene expression in multiple tissues of silkworm larvae on the 3rd day of the 5th instar [23], we observed that 79 of the predicted candidate BmSoxE targets were expressed in at least one tissue in silkworm larvae, 25 of which were down-regulated (Fig. 6 and Online Resource 7) while 54 were up-regulated (Fig. 7 and Online Resource 8). Notably, 68 candidate BmSoxE targets, including 19 down-regulated genes and 49 up-regulated genes, were expressed in the silkworm gonad. Five of the down-regulated candidate targets were specifically expressed in the silkworm gonad, three of which were annotated as *TCF25*, *TACC*, and as *BmSoxE* itself. Among the 10 up-regulated candidate targets with gonad-specific expression, seven were annotated as *His2B*, *N*-*acetylneuraminate lyase*, *EFTUD1*, *Bsf*, *TXNDC12*, *Hsp19.5*, and *RluA*-*1*.

## Discussion

SoxE transcription factors have been identified as key regulators of multiple critical cellular processes, particularly during testis development in animals [1, 13]. Similar to Sox proteins from other groups, SoxE proteins play key roles by regulating the transcription of their binding targets. Although several targets of the SoxE proteins have been reported, such findings have been limited in mammals, mainly coming from mice. Among insects, SoxE has been shown to be essential for testis development in *D. melanogaster* [19] and to be highly expressed in the silkworm gonad [14]. However, the signaling pathways of insect SoxE protein, particularly its binding targets, are largely unknown.

In this study, we focused on the *BmSoxE* gene from the silkworm and performed an RNAi analysis in ovary-derived BmN4-SID1 cells to characterize the roles of BmSoxE in cell proliferation and to identify its candidate targets. Intriguingly, the RNAi-mediated silencing of *BmSoxE* expression in BmN4-SID1 cells suppressed cell proliferation and induced G1 cell cycle arrest. These results are similar to the effects of RNAi knockdown of other genes involved in cell cycle progression, such as *livin* in human osteosarcoma cells [33], *cyclin*-*dependent kinase 6* in medulloblastoma cells [34], *Bmi*-*1* in laryngeal carcinoma cells [35], *NANOG* in breast cancer cells [36], and *EZH2* in colon cancer cells [37]. Therefore, the inhibition of cell proliferation observed in BmN4-SID1 cells following *BmSoxE* RNAi may derive from a disruption of cell cycle progression.

Genome-wide microarray analyses revealed that the expression levels of 320 genes were significantly down- or up-regulated after *BmSoxE* RNAi in silkworm BmN4-SID1 cells, indicating that BmSoxE also functions as a positive or negative regulator of gene expression. Importantly, 108 differentially expressed genes were predicted to possess at least one conserved binding motif for the HMG box of Sox proteins in their upstream UTR regions, suggesting that these genes are candidate binding targets for the BmSoxE protein. In previous reports in rats or mice, many binding targets have been identified or predicted for three members of the E group Sox subfamily, Sox8, Sox9, and Sox10, based on their conserved binding motifs [10–12]. However, additional evidence should be acquired from ChIP-seq (chromatin immunoprecipitation sequencing) together with in vitro EMSA (electrophoretic mobility shift assay) analyses to determine the actual binding properties of the BmSoxE protein in the transcriptional regulation of its candidate targets in the future.

Given that BmSoxE is involved in the regulation of cell proliferation, we speculated that candidate BmSoxE targets are likely involved in the regulation of cell proliferation. As expected, we noted that the homologs of many candidate targets of the BmSoxE protein have been demonstrated to modulate cell proliferation in other organisms. For example, as listed in Table 1, there were 10 genes involved in the regulation of cell cycle progression or DNA replication, including *MU2* [38], *Glypican*-*1* [39], *Gadd45* [40], *Mcm10* [41–43], *TACC* [44], *Peroxidasin* [45], *Nudt1* [46], *SEPW1* [47], *Cyt*-*b5* [48], *Orb2* [49], and *Vein* [50, 51]. In addition, two genes were associated with the DNA repair process, namely *MU2* [38] and *Gadd45* [40]. Seven genes were related to the regulation of cell apoptosis/death, which included *Mad2* [52], *DENN* [53], *Gadd45* [54], *GlcT*-*1* [55], *Ced*-*6* [56], *tensin* [57], and *Cyt*-*c*-*p* [58]. Furthermore, *Tsp2A* and *TBC1D7* are responsible for the control of cell growth [59, 60]. Taken together with the above-mentioned functional cues obtained through homologous annotation, the expression changes observed in these cell cycle-related genes following *BmSoxE* RNAi indicated not only that that the BmSoxE protein lies in a pathway upstream of the regulation of the cell cycle, but also that these candidate BmSoxE targets may cooperatively contribute to the suppression of cell proliferation in BmN4-SID1 cells. Therefore, it is worth clarifying how BmSoxE regulates these candidate targets to control the cell cycle and cell proliferation in further studies.

Cell proliferation is generally mediated by multiple cell cycle events [61, 62]. Our results showed that two core regulators of cell cycle, Gadd45 and Mcm10, were included in the candidate BmSoxE target set and were significantly down-regulated by *BmSoxE* RNAi. Previous reports have demonstrated that Gadd45 is highly expressed in the G1 phase of the cell cycle [63] and can interact with the p21 protein, which mediates G1 arrest [64, 65], or with the PCNA protein, which is required for DNA replication and repair [65, 66]. Mcm10 has been shown to be associated with the control of DNA replication [42, 43]. There is a possibility that BmSoxE may control cell proliferation via the modulation of Gadd45 and Mcm10 in the cell cycle in BmN4-SID1 cells. However, the majority of the core regulators of the cell cycle were excluded from the collection of candidate BmSoxE targets obtained in the current study. Several core regulators associated with cell cycle progression and DNA replication displayed moderately down- or up-regulated expression after *BmSoxE* RNAi (Online Resource 5 and Online Resource 6). We speculate that this nonsignificant deregulation may also contribute to the *BmSoxE* RNAi-induced inhibition of cell proliferation. Our next aim is therefore to decipher the interactions among BmSoxE, candidate BmSoxE targets, and cell cycle regulators during cell proliferation, which may provide a comprehensive understanding of the function and regulation of the BmSoxE protein.

*SoxE* expression is enriched in the gonad of animals and predominately regulates gonad development and sex determination [1, 13]. Based on published microarray data [23], we found that 15 candidate BmSoxE targets were specifically expressed in silkworm gonad (Figs. 6, 7). Notably, the homologs of several gonad-specific candidate targets have been characterized as being associated with genital events in other animals. For instance, the mouse *TACC3* gene is abundantly expressed in adult testis and ovarian cells during gonad growth and development [67]. The expression of the *His2B* gene is regulated by the argonaute protein CSR-1 in the *C. elegans* gonad [68]. BSF contributes to mRNA stabilization of the *bicoid* gene, which encodes a transcription factor that activates the expression of zygotic genes during *D. melanogaster* embryogenesis [69, 70]. Therefore, these findings related to candidate BmSoxE targets will help us to elucidate the molecular mechanisms underlying gonad development and sex determination in the silkworm.

In conclusion, we confirmed that RNAi-based silencing of the expression of the transcription factor *BmSoxE* inhibited cell proliferation in silkworm BmN4-SID1 cells. Many genes that were differentially expressed following *BmSoxE* RNAi contained conserved binding sites for the Sox protein and were predicted to be candidate binding targets for the BmSoxE protein. Furthermore, some candidate BmSoxE targets may be associated with the regulation of cell proliferation. Our findings should be useful for deciphering the functions and signaling pathways of insect SoxE in the regulation of cell cycle progression.

## Electronic supplementary material

Below is the link to the electronic supplementary material.

Online Resource 1 List of primers used in this study

Online Resource 2 List of genes showing significantly altered expression levels after *BmSoxE* RNAi in silkworm BmN4-SID1 cells

Online Resource 3 Subcellular distribution and mRNA expression of *BmSoxE* in silkworm BmN4 cells. (a) The subcellular localization of the transiently expressed Venus-BmSoxE fusion protein in silkworm BmN4 cells was determined based on fluorescence (green), and nuclear DNA was counterstained with DAPI (blue). The Venus-BmSoxE fusion protein was located only in the nucleus. As a control, the localization of the parental construct Venus-Dest was examined, and it was found to be expressed in both the cytoplasm and nucleus. Scale bar, 10 μm. (b) RT-PCR detection of mRNA expression of silkworm *BmSoxE* in BmN4 cells

Online Resource 4 GO annotation of all genes expressed in BmN4-SID1 cells after *BmSoxE* or *EGFP* RNAi

Online Resource 5 BmSoxE RNAi-mediated nonsignificant expression alteration of core regulators related to cell cycle progression

Online Resource 6 BmSoxE RNAi-mediated nonsignificant expression alteration of core regulators related to DNA replication

Online Resource 7 List of candidate BmSoxE targets that were down-regulated after *BmSoxE* RNAi in silkworm BmN4-SID1 cells and were expressed in silkworm larval tissues, including those showing gonad-specific expression

Online Resource 8 List of candidate BmSoxE targets that were up-regulated after *BmSoxE* RNAi in silkworm BmN4-SID1 cells and were expressed in silkworm larval tissues, including those showing gonad-specific expression
