# Supplementary material for: RNAi silencing of the SoxE gene suppresses cell proliferation in silkworm BmN4 cells
Source: Mol Biol Rep. 2014 Apr 11;41(7):4769–81. doi: 10.1007/s11033-014-3348-6 (PMC4066180; doi:10.1007/s11033-014-3348-6)
Supplement: Supplementary file 3 — Subcellular distribution and mRNA expression of BmSoxE in silkworm BmN4 cells. (a) The subcellular localization of the transiently expressed Venus-BmSoxE fusion protein in silkworm BmN4 cells was determined based on fluorescence (green), and nuclear DNA was counterstained with DAPI (blue). The Venus-BmSoxE fusion protein was located only in the nucleus. As a control, the localization of the parental construct Venus-Dest was examined, and it was found to be expressed in both the cytoplasm and nucleus. Scale bar, 10 μm. (b) RT-PCR detection of mRNA expression of silkworm BmSoxE in BmN4 cells [file 11033_2014_3348_MOESM3_ESM.doc]

Molecular Biology Reports

**RNAi silencing of *SoxE* gene suppresses cell proliferation in the silkworm BmN4 cells**

Ling Wei, Zhiqing Li, Daojun Cheng, Takahiro Kusakabe, Minhui Pan, Jun Duan, Yonghu Wang, Cheng Lu ^*^

^*^ Correspondence

State Key Laboratory of Silkworm Genome Biology, Southwest University, Chongqing, China.

E-mail: lucheng@swu.edu.cn

**Online Resource 3**

**ESM (Electronic Supplementary Material) 3**

**Online Resource 3** Subcellular distribution and expression of *BmSoxE* in silkworm BmN4 cells. (a) Subcellular localization of transiently expressed Venus-BmSoxE fusion protein in silkworm BmN4 cells was determined by fluorescence (green) and the nuclei DNA was counterstained with DAPI (blue). The Venus-BmSoxE fusion protein was located only in the nucleus. As a control, the localization of parental construct Venus-Dest was evenly expressed both in the cytoplasm and nucleus. Scale bar, 10 μm. (b) RT-PCR detection of mRNA expression level of silkworm *BmSoxE* in BmN4 cells.
