# Supplementary material for: RNAi silencing of the SoxE gene suppresses cell proliferation in silkworm BmN4 cells
Source: Mol Biol Rep. 2014 Apr 11;41(7):4769–81. doi: 10.1007/s11033-014-3348-6 (PMC4066180; doi:10.1007/s11033-014-3348-6)
Supplement: Supplementary file 4 — GO annotation of all genes expressed in BmN4-SID1 cells after BmSoxE or EGFP RNAi [file 11033_2014_3348_MOESM4_ESM.doc]

Molecular Biology Reports

**RNAi silencing of *SoxE* gene suppresses cell proliferation in the silkworm BmN4 cells**

Ling Wei, Zhiqing Li, Daojun Cheng, Takahiro Kusakabe, Minhui Pan, Jun Duan, Yonghu Wang, Cheng Lu ^*^

^*^ Correspondence

State Key Laboratory of Silkworm Genome Biology, Southwest University, Chongqing, China.

E-mail: lucheng@swu.edu.cn

**Online Resource 4**

**ESM (Electronic Supplementary Material) 4**


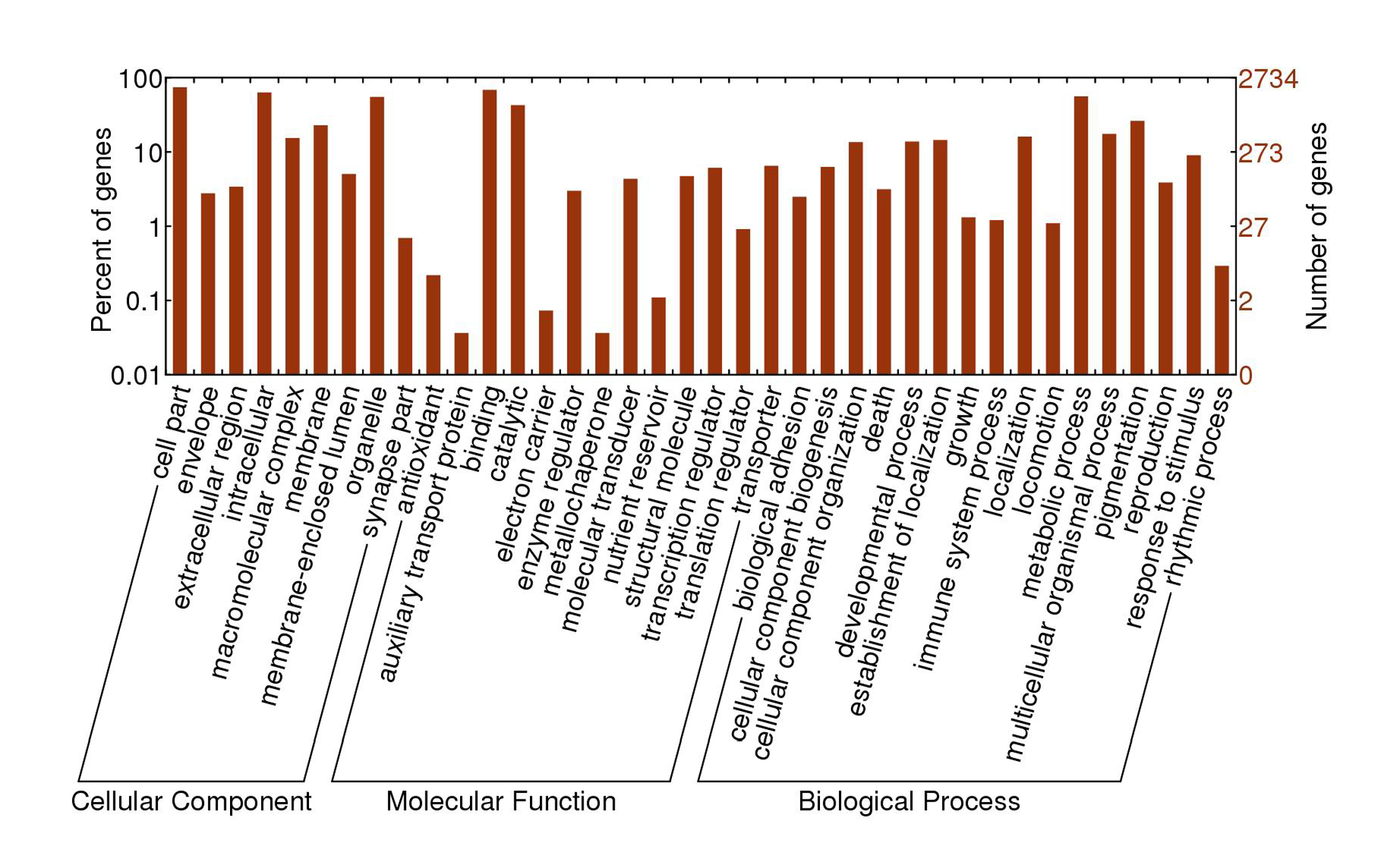


**Online Resource 4** GO annotation of all 6,275 genes expressing in BmN4-SID1 cells after *BmSoxE* RNAi and *EGFP* RNAi.
